# Attention to memory: Asymmetric enhancement of mnemonic features in auditory working memory

**DOI:** 10.3758/s13423-026-02991-8

**Published:** 2026-09-10

**Authors:** David Peita, Sung-Joo Lim

**Affiliations:** Department of Psychology, Binghamton University, 4400 Vestal Parkway E, Binghamton, NY 13902 USA

**Keywords:** Auditory working memory, Attention to memory, Auditory objects, Memory precision

## Abstract

Models of auditory attention propose that multiple sound features are integrated into a perceptual object and that selective attention enhances the fidelity of these object-based representations. Similar attentional mechanisms appear to operate on representations held in auditory working memory, yet evidence has largely focused on a single feature. Thus, it remains unknown whether redirecting attention to an auditory memory object enhances multiple, distinct features bound to that object, or whether such enhancement varies across features depending on modality-specific biases or prior knowledge of the to-be-probed feature. Here, we investigated whether retrospective attention improves the mnemonic fidelity of two features—pitch and spatial location—associated with speech objects using psychophysical modeling. Participants encoded two syllables varying in both pitch and location and received valid (vs. neutral) retroactive cues during retention. They then judged a change in one of the two features of the cued syllable. Two groups performed the same task but differed in whether they knew which feature would be probed. Across groups, valid retro-cues facilitated faster judgments of both features, with larger benefits when the to-be-probed feature was known. However, cue-related improvements in accuracy and perceptual precision emerged only for pitch, not spatial location, regardless of feature awareness. This asymmetry suggests that retrospective attention does not uniformly enhance all features of auditory memory objects, but favors auditory identity-related features, such as pitch, over spatial information. These findings demonstrate that attention and memory interact differently across feature dimensions, revealing limits on how object-based attention operates over auditory working memory.

**Supplementary Information:**

The online version contains supplementary material available at https://doi.org/10.3758/s13423-026-02991-8.

## Introduction

Sounds unfold rapidly over time and often overlap with one another. To interpret such complex and transient sensory signals, listeners must construct internal representations of sounds and maintain them in memory. For instance, navigating a crowded space requires listeners to form stable “auditory objects”—representations that integrate multiple features such as pitch, timbre, and location (Griffiths & Warren, 2004; Shinn-Cunningham, 2008). Maintaining these objects in short-term (or working) memory is crucial for coherent perception and goal-directed behavior (Alain et al., 2009; Wilsch & Obleser, 2016; Zimmerman et al., 2016). Yet working memory is inherently limited in capacity, posing a challenge for maintaining accurate and perceptually precise memory representations (Bays & Husain, 2008; Ma et al., 2014).

Conceptualizing working memory as a resource pool, selective attention plays a crucial role in overcoming these limitations by flexibly allocating mnemonic resources to objects (Bays, 2015; Ma et al., 2014). In particular, retrospective attention—attention redirected to items held in memory—not only facilitates fast and accurate recall of cued items (Backer & Alain, 2014; Oberauer & Hein, 2012; Pertzov et al., 2013; Souza & Oberauer, 2016), but also enhances the representational precision of attended memory items (Griffin & Nobre, 2003; Kumar et al., 2016; Lepsien & Nobre, 2007; Lim et al., 2015). However, it remains unknown whether this mnemonic representational enhancement spreads to multiple features bound within the same auditory object.

This question is directly relevant to the object-based attention framework, which posits that attention directed to an object enhances the processing of all its features (Duncan, 1984; Shinn-Cunningham, 2008). In the auditory domain, studies show that attending to one feature (e.g., pitch) enhances the processing of other features (e.g., location) within the same object (Dyson & Ishfaq, 2008; Shinn-Cunningham, 2008). Consistent with this, auditory retrospective attention improves the recall precision of attended objects for features like pitch (Kumar et al., 2016) even when attention is redirected to more abstracted linguistic representations (e.g., syllables; Lim et al., 2015). These benefits come at the cost of increased demands to reallocate cognitive resources to cued items, reflected in enhanced neural alpha oscillatory power (Backer et al., 2015; Lim et al., 2015) and engagement of domain-general attentional networks (Backer et al., 2020; Joseph et al., 2015; Lim et al., 2021). However, because these studies have mostly probed a single feature (Kumar et al., 2016; Lim et al., 2015), it remains unclear whether retrospective attention flexibly enhances the mnemonic precision of multiple features integrated within the attended auditory object, or whether such enhancements are feature-specific.

A feature-specific precision enhancement from retrospective attention may reflect either modality-specific biases or may be specific to the feature receiving directed attention. Although auditory working memory maintains multiple object-defining features (e.g., pitch and location) in an integrated fashion (Backer et al., 2015; Dyson & Ishfaq, 2008; Fischer et al., 2024), several previous studies (Delogu et al., 2014; Kubovy & Van Valkenburg, 2001; Maybery et al., 2009) have suggested an asymmetry in how these discrete features are processed and stored. Specifically, these studies have shown that auditory working memory preferentially encode and maintain identity-defining features like pitch, whereas spatial information may serve as a nonprimary feature that is less robustly retained. Given that pitch and spatial features are maintained via separate cortical pathways (Ahveninen et al., 2006; Alain et al., 2001; Maeder et al., 2001; Rauschecker & Tian, 2000), there may be inherent constraints on how flexibly reorienting attention within auditory memory can enhance the precision of different features.

Furthermore, according to the modality appropriateness hypothesis, different sensory modalities are specialized for processing particular types of information (Welch & Warren, 1980). Spatial information, for example, is more preferentially processed within the visual than auditory modality (Alais & Burr, 2004; Mahar et al., 1994; Noyce et al., 2016). Supporting this idea, neuroimaging studies further show that spatial processing—whether for visual or auditory stimuli—recruits visual-biased regions of the lateral frontal cortex that are implicated in attention and working memory functions (Michalka et al., 2015; Noyce et al., 2017). These findings suggest that there may be separate mnemonic resources defined by the sensory modality and their corresponding preferential features (Bays et al., 2011; Pasternak & Greenlee, 2005), which could constrain how flexibly retrospective auditory attention can enhance the fidelity of multiple features of an attended object. Thus, although both pitch and spatial features of auditory objects are maintained in working memory (Czoschke et al., 2021; Fischer et al., 2024), the effectiveness of retrospective attention in highlighting these features may depend on the modality-specific salience, and it may require directed feature-level attention to precisely recall the less salient feature.

To address this question, we employed a delayed recall of syllable-feature discrimination task adapted from prior auditory working memory paradigms (Alavash et al., 2018; Lim et al., 2015, 2018, 2021). Participants encoded two discrete syllables varying in pitch and spatial location and received a valid (or neutral) retro-cue to direct attention to one syllable prior to judging a change in either the pitch or location feature. Recall precision for each feature was estimated using psychophysical modeling (Bays & Husain, 2008; Lim et al., 2015; Murray et al., 2013; Zhang & Luck, 2008). To examine whether attending to one task-relevant feature influences these effects, one group of participants performed the task in which trials within each block probed only one consistent feature, which allowed them to discover the to-be-probed feature in advance, whereas the other group performed the same task where both features were probed equally within each block, requiring them to retain both features until hearing a probe.

We hypothesized that if the pitch and spatial features are tightly integrated in an object-based form, retrospective attention to a syllable object should equally enhance recall precision for both features. Conversely, if the retrospective attentional benefit is feature-specific and is constrained by auditory modality preferences in how different features are processed and represented, and/or depends on explicit attention to a task-relevant feature, we expected greater precision benefit for pitch than location, but this difference between the two features would depend on listeners’ awareness of the to-be-probed feature.

## Materials and methods

### Participants

We estimated the sample size based on previous studies. Based on prior work using a similar auditory retro-cueing task (Lim et al., 2015, 2018, 2021), a sample size of 17 participants would provide at least 80% power to detect within-subjects effects of retro-cues on enhancing syllable-pitch precision. Other studies examined listeners’ ability to detect changes in the spatial and pitch features of auditory working memory objects using a match–nonmatch paradigm (Delogu et al., 2014; Fischer et al., 2024) based on the effect sizes reported in these studies for response times and accuracy measures, sample sizes between seven to 20 participants would achieve > 80% power. Based on these estimates, we recruited 20 participants for each group.

Forty young adults (27 women, mean age = 20.13 ± 3.53 years, range: 18–34 years) participated in the current study. One additional participant took part in the experiment but was excluded from the data analyses due to overall performance below chance (50%). Twenty participants were assigned to the feature-predictable group, in which one consistent feature was probed per each task block; the remaining participants were assigned to the feature-unpredictable group, in which either feature was randomly probed, requiring them to retain both features until hearing the probe.

All reported normal hearing and normal or corrected-to-normal vision. None reported having histories of neurological or speech, language, and hearing disorders. Participants gave written informed consent. The Institutional Review Board of Binghamton University approved the study protocol. All participants were recruited from Binghamton University and received course credit or monetary compensation ($15/hour) for participation.

### Stimuli

The auditory stimuli were drawn from previous studies (Alavash et al., 2018; Lim et al., 2015, 2021). In brief, listeners heard natural recordings of two syllable sounds (/da/and/ge/) in the syllable feature discrimination task. Each syllable category consisted of six tokens, which served as sample sounds for which to discriminate featural changes occurring against probe sounds. Each syllable token had a combination of two features, spectral pitch (i.e., the fundamental frequency; F0) and a spatial location. The F0s of the syllable tokens ranged 122.11–213.91 Hz for/da/and 129.71–233.88 Hz for/ge/categories, respectively. The spatial location of feature was generated by creating interaural time difference (ITD) of 7.4 and 15.2 ms, which roughly corresponds to 30° and 60° to the left and to the right along the azimuth from center. Because spatial acuity in audition varies as a function of azimuth—with better acuity near the midline than in the periphery (Mills, 1958)—these starting azimuths were selected to fall within a relatively stable localization range (Perrott & Saberi, 1990). This range also overlaps with the azimuths tested in a prior auditory space recall study employing ITD manipulations over headphones (i.e., 13°–64° azimuths; Czoschke et al., 2021), while still providing sufficient dynamic range to systematically generate subsequent spatial shifts within the same hemispace (i.e., not crossing the 0° midline).

For each to-be-probed sample token, corresponding probe stimuli were generated by parametrically manipulating fundamental frequency (F0) and spatial location. These features were simultaneously varied in six steps, ±1, ±2.5, and ±4.5 times each individual’s 50% detection threshold obtained from adaptive tracking procedures (see below), relative to the sample token presented during syllable encoding. These step sizes were chosen to sample stimulus changes along a log-scale continuum, allowing us to capture participants’ response patterns both near the threshold and further along the psychometric function. This ensured a reliable estimation of the perceptual precision parameter via logistic fitting (Kingdom & Prins, 2016). To illustrate, on a given trial, if a participant’s pitch threshold was 0.5 semitones and spatial location threshold was 8°, applying the same ± 2.5 multiplier would result in a 1.25-semitone change in pitch and a 20° shift in spatial location. 

F0 manipulation was accomplished using the frequency extraction and resynthesizing functions in STRAIGHT (Kawahara, 2006). Spatial location of syllables was manipulated by adjusting the interaural time difference (ITD) using the Woodworth model (Aaronson & Hartmann, 2014). To prevent spatial location shifts from crossing the midline (0°) or exceeding 90° at probe, which could make them more perceptually salient, the starting location of the sample token was adjusted to ensure all probed spatial locations fell between 0° and 90° on each side. This adjustment was necessary for only three of the 40 participants, for whom the starting locations were altered on an average of 16.9% of trials.

All tokens were 200 ms in duration and edited to have 3 ms linear onset and 30 ms offset ramps. All tokens were normalized to equivalent room-mean-squared amplitude (65 dB SPL).

### Task design

All participants performed a syllable feature-discrimination task, adapted from the previously established auditory retroactive cueing paradigm by Lim et al. (2015). We used a 2 × 2 design to manipulate the within-subjects factors of 2 *retro-cues* (valid vs. neutral) × 2 *to-be-discriminated features* (pitch vs. spatial location) of a syllable in the experiment. Two groups of participants differed only in the predictability of the to-be-discriminated feature within each block; in the feature-predictable group, trials within a block probed only a single feature (either pitch or spatial location), whereas in the feature-unpredictable group, trials probing both features were randomly intermixed within each block (see below).

**Fig. 1:**
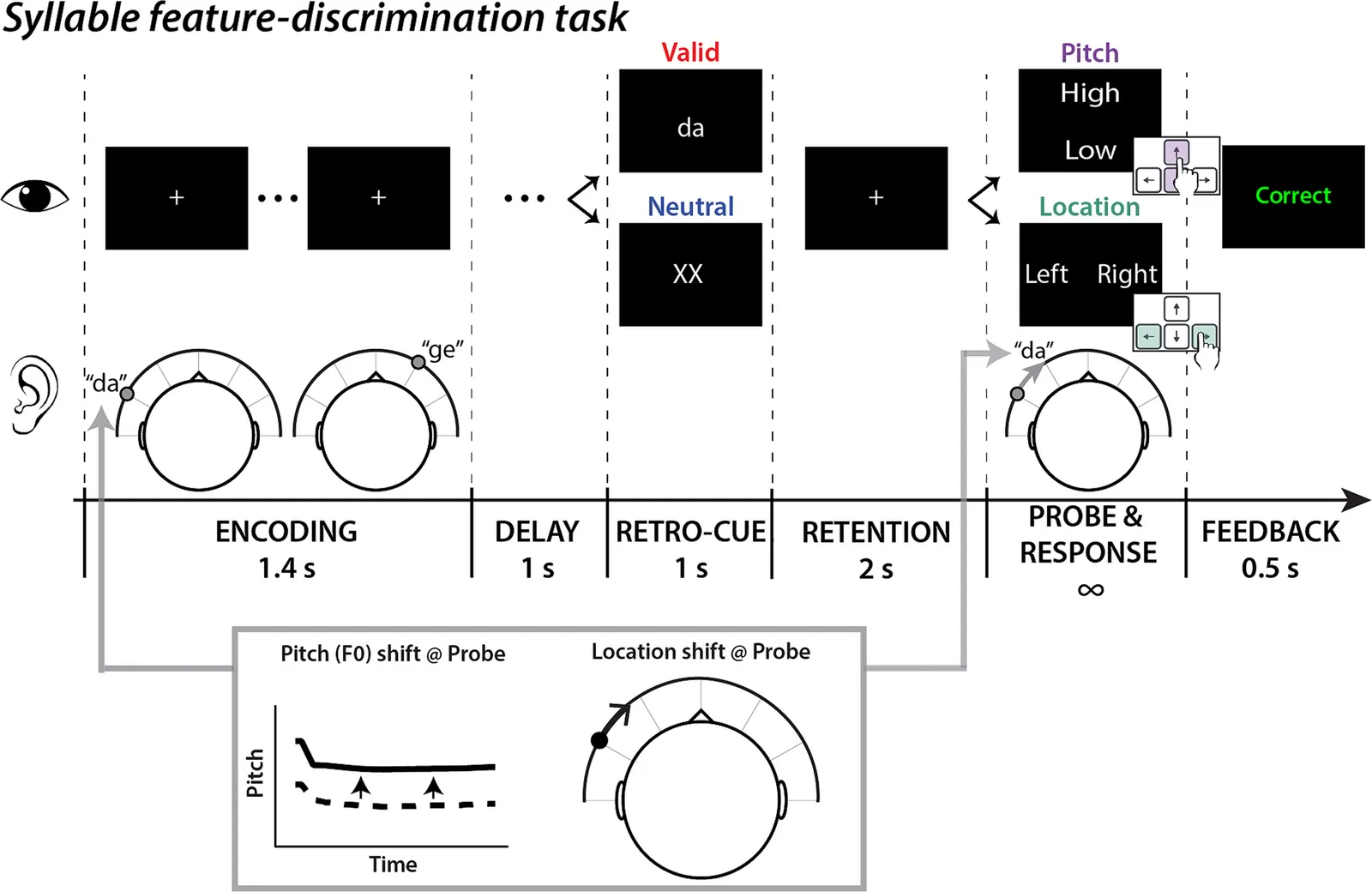
Trial structure of the syllable feature-discrimination task. On each trial, participants heard two syllables (200 ms each), separated by a 1-s silence. Two syllables were presented from two of four possible spatial locations chosen pseudo-randomly on each trial (unless adjustment was required; see the Materials and Methods section). The two sample syllables were always presented on opposite sides (one left, one right) to ensure the spatial judgment required comparing the same syllable object, rather than using the nonprobed syllable as an additional reference. All four locations were equally probed throughout the experiment. During a 4-s memory-retention interval, a visual retro cue was presented for 1 s. After an additional 2-s delay, a probe syllable token was then presented, and participants judged the probe on one of the two feature dimensions, as indicated on the response screen. The highlighted arrow keys were used for the corresponding response prompt. For the feature-predictable group, each task block probed only one feature (either pitch or location). For the feature-unpredictable group, both features were equally probed within each block, requiring participants to monitor changes in both features. Trial types are labeled in bold text and the corresponding highlighted response keys in the figure are shown for illustration purposes only

Figure 1 illustrates the trial structure. In each trial, participants heard two distinct auditory syllables (i.e., /da/and/ge/, each 0.2 s in duration) presented in a random order, separated by 1 s of silence. The two syllables were always presented in opposite hemispaces (one on the left, the other on the right) at starting azimuths of either 30° or 60° relative to the midline. After a 1-s delay, a visual retro-cue was presented for 1 s. After an additional 2-s delay, one of the syllables heard during encoding was presented as a probe with both its pitch (F0) and spatial location varied by the identical threshold multiplier relative to the encoding phase. Upon hearing the probe, a response screen appeared indicating which of the two featural changes listeners were asked to discriminate against the same syllable category sound presented during encoding. Participants responded using arrow keys on a keyboard. A response screen appearing as “HIGH, LOW” instructed listeners to judge whether the probe syllable pitch was higher or lower compared with the corresponding syllable presented during encoding using the up/down arrow keys; a response screen appearing as “LEFT, RIGHT” prompted listeners to judge whether the probe syllable shifted left or right relative to the same syllable presented during encoding using the left/right arrow keys. For example, if participants heard a /da/ syllable as a probe, then either the pitch or spatial location of this probe—informed by the response screen—should be compared to the /da/ sound presented in the encoding phase. To ensure an intuitive and semantically congruent mapping, the text on the response screen was spatially mapped to the physical layout of the arrow keys; this configuration remained constant for all participants. After providing a response to a given probe, participants received visual feedback for 500 ms.

During the memory-retention phase, participants were given two types of retro-cues. The “valid” retro-cues indicated which of the two syllables would be probed. In these trials, participants were presented with a written “da” or “ge” on the screen for 1 s and heard a probe sound from the corresponding syllable category. In the “neutral” retro-cue trials, participants only saw “xx” written on the screen, indicating that either of the two encoded syllables could be probed. As such, participants had to retain information about both syllables in working memory until hearing a probe syllable.

Participants completed a total of 192 trials, given across eight blocks of 24 trials. The trial organization within each block differed between the two participant groups. The feature-unpredictable group was asked to judge the changes in the two feature dimensions (pitch vs. spatial locations) randomly and equally in each block. Thus, listeners in this group were unaware which of the two features was task relevant for the given trial, and they had to maintain both features of sample syllables until they received the response screen. In contrast, participants in the feature-predictable group performed the same task, but trials within each block probed only one feature (i.e., either pitch or spatial changes), and the to-be-probed feature alternated across blocks. Thus, listeners in the feature-predictable group could have attended only one feature of the syllable to perform the task.

For all participants, the two retro-cues were equally presented and both syllables presented during encoding were equally probed within each block. The four starting spatial locations were probed equally often throughout the experiment. Step sizes, syllable identities, and directions of spatial change were counterbalanced across starting azimuths and hemispaces to assess the effects of interest while minimizing potential confounding effects of eccentricity.

### Adaptive tracking for individual adjustment of auditory stimuli

To calibrate stimulus changes used in the main experimental task while accounting for potential individual differences in sensitivity to pitch and spatial location changes of sounds, we estimated individual’s change-detection threshold for each feature. Prior to the main experiment, participants completed three blocks (32 trials per block) of an auditory feature discrimination task in each dimension.

We utilized a roving adaptive tracking procedure in which participants heard pairs of sounds that varied across trials (i.e., without a fixed reference). On each trial, two stimuli were separated by a 5-s interstimulus interval (ISI). Although these task parameters are known to significantly increase discrimination thresholds (e.g., Ahissar et al., 2006; Amitay et al., 2005; McPherson & McDermott, 2020; Wisniewski & Tollefsrud, 2023), this design was chosen to accommodate the demands of the primary task. A one-up one-down adaptive tracking was used to estimate the change detection threshold at approximately 50% performance for each feature (Levitt, 1971). No feedback was provided.

#### Pitch threshold tracking

Pitch discrimination thresholds were estimated using pure tones. On each trial, participants heard two tones (200 ms in duration) separated by 5 s and judged whether the second tone was higher or lower in pitch than the first. The initial pitch separation was 3 semitones and was adjusted to a minimum step size of 0.0625 semitones towards the end of the block. The frequency of the first tone was randomly drawn from 100–300 Hz, and the second tone frequency was adjusted to be higher or lower from the first tone according to the pitch separation defined for that trial.

#### Location threshold tracking

Speech syllables (/da/ or /ge/) were used to estimate individuals’ thresholds for detecting auditory spatial location changes. On each trial, participants heard a syllable twice, separated by 5 s, with the second syllable shifted in spatial location relative to the first. Participants judged whether the location of the second syllable shifted leftward or rightward along the azimuth. The initial spatial separation between the two sounds was 30° and was adjusted in steps of 8° to minimum of 2°. On each trial, the first sound was randomly presented at one of four locations (either 30° or 60° left or right from the center, matching the starting azimuths used in the primary task), and the second sound was presented either leftward or rightward based on the spatial separation determined for the corresponding trial. Note that we ensured that the location shifts between the two sounds never crossed the center/midline or exceeded 90° on either side.

Change detection threshold for each feature was determined by averaging the thresholds obtained in three blocks (i.e., the average of the last six reversals per block). On average, the 50% detection thresholds across participants were 0.447 ± 0.251 semitones for pitch and 7.68° ± 4.02° for spatial location. Individual thresholds were subsequently used to create the probe stimuli used in the main task.

### Procedure

Upon the completion of the adaptive tracking procedures, all participants went through a short practice of the syllable feature discrimination task (20 trials) with easily detectable pitch and location changes (× 4.5 of their own thresholds), which was the maximum magnitude used in the main experiment. Trials of the 2 *retro-cue* × 2 *feature-type* conditions were intermixed during practice. If the participants’ performance was below 70%, the practice session was repeated (out of 40 participants, six participants repeated the practice once and two repeated it twice). Next, participants went through the syllable feature-discrimination task.

All experimental trials were controlled with Psychtoolbox (MATLAB R2017a; Brainard, 1997). Auditory stimuli were delivered via headphones (Sennheiser 280-HD Pro) at 65 dB sound pressure level (SPL). The experiment was conducted in a double-walled sound-attenuated booth and visual stimuli were presented on a BenQ MOBIUZ EX2510 24.5-inch monitor.

### Data analysis

Response time (RT) of the correct trials and response accuracy served as overall behavioral performance measures. RT was measured from the onset of the probe syllable. To approximate normality, RTs were log-transformed prior to analysis. Any outlier log-transformed RTs exceeding three standard deviations (*SD*s) from an individual participant’s average RT were excluded. In addition, we excluded an average of 0.8% of trials (i.e., 1.5 trials per participant), in which the spatial location of the probe sound fell outside the predefined spatial boundaries (i.e., crossed the midline or exceeded ± 90°) prior to data analysis. Response times (RTs) were analyzed using a linear mixed-effects model, whereas trial-wise accuracy data were separately analyzed using a generalized linear mixed-effects model with a binomial error distribution and logit link function (*lme4* in R Version 3.3.3). The mixed-effects models included within-subjects fixed factors of *retro-cue* (valid vs. neutral) and *feature type* (pitch vs. location), and the between-subjects factor of *feature-predictability* (predictable vs. unpredictable). The random-effects term included by-participant intercepts and slopes for all within-subjects factors. Deviation-coded (sum) contrasts were applied to all of the fixed factors. Significance of fixed factors was determined by a Type III analysis of variance (ANOVA) at an α = 0.05, with *p* values based on the Satterthwaite approximation of the degrees of freedom (*lmerTest* package in R). Significant effects found from the ANOVA were followed by post-hoc pairwise tests using *emmeans* in R.

Furthermore, we also quantified a more fine-grained perceptual precision measure in detecting the pitch and spatial location changes of auditory syllables. We used the psychophysical modeling approach, which has been widely used in psychophysics research across various domains (Bays & Husain, 2008; Herbst & Obleser, 2019; Lim et al., 2015, 2018; Murray et al., 2013; Zhang & Luck, 2008). To measure individual participant’s perceptual precision in detecting the change in each feature dimension, we separately fitted each participant’s response patterns to the varying levels of pitch and location change occurred at the probe relative to the sample syllable heard during encoding (Fig. 3A). We used a nonlinear least squares curve-fitting procedure (*lsqcurvefit* function from MATLAB) with a logistic (sigmoid) function,

$$y = \frac{1}{1+ {e}^{-k(x-m)}}$$

where *y* indicates the proportion of “high” or “right” responses, *x* indicates the step sizes of F0 or spatial degree change at the probe relative to the encoded syllable in working memory, *k* indicates the slope, and *m* indicates the inflection point of the logistic function. The inflection point (*m*) provides an estimate of response bias. The slope (*k*) estimates the perceptual precision in the pitch or location change detection: the steeper the slope, the greater the perceptual precision (Fig. 3A).

## Results

**Fig. 2:**
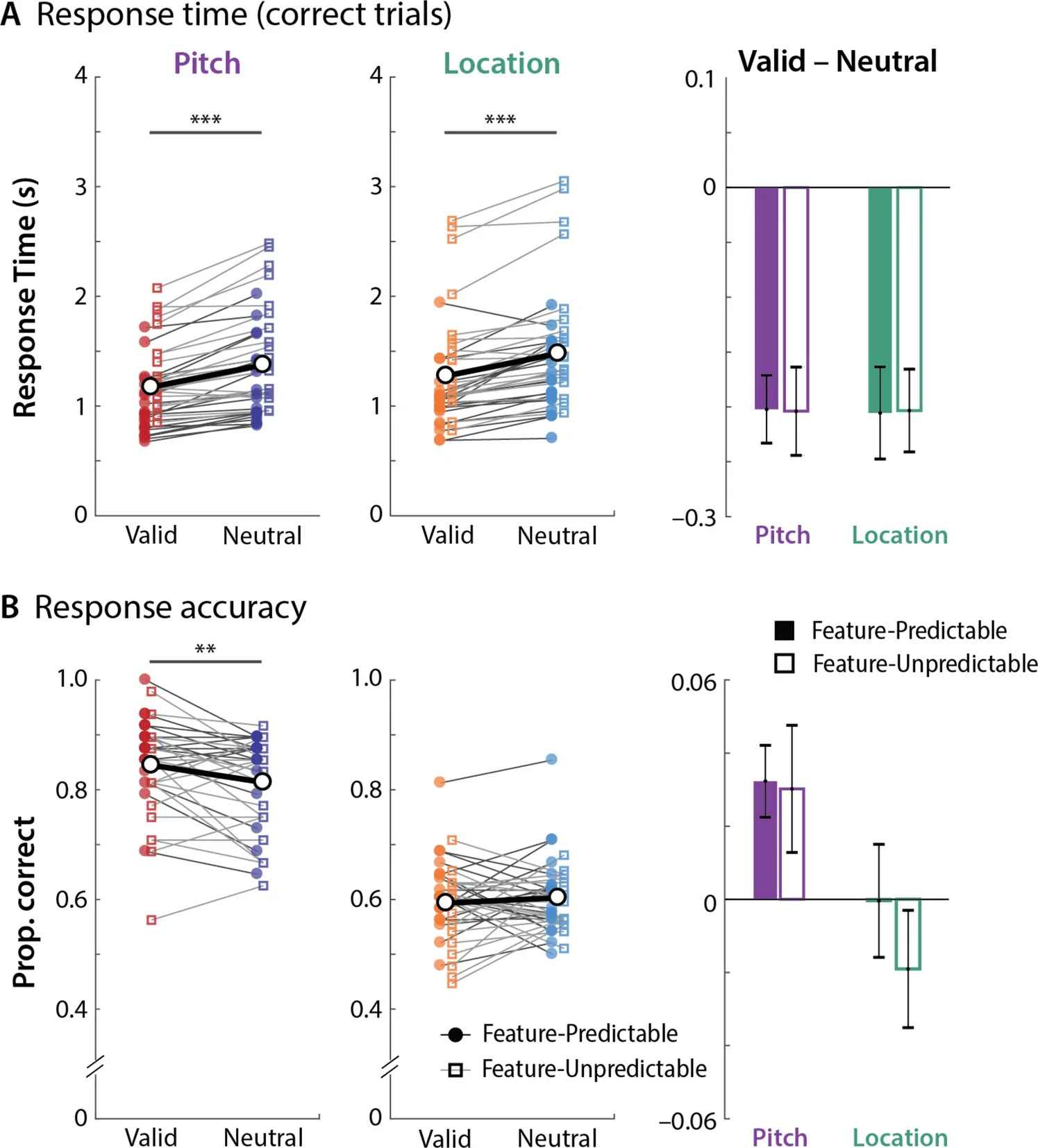
Behavioral performances of the valid and neutral retro-cue trials in each feature condition. Response times (RTs) of correct trials **(A)** and response accuracy **(B)**. (Left) Points connected by thin lines represent individual participants’ average performances in the respective measure. Filled circles represent the feature-predictable group participants, open squares represent the feature-unpredictable participants. Large circles with a bold line represent grand averages of the respective measures in each feature condition. (Right) The mean performance benefits from valid (vs. neutral) retro-cues for each feature type. The filled and open bars represent the measures of the participants who belong to the feature-predictable and feature-unpredictable groups, respectively. The error bars represent ±1 standard error of the mean (*SEM*). **p < 0.01, ***p < 0.001. (Color figure online)

First, we evaluated the effects of retro-cues (valid vs. neutral) on response times (RTs) for judging pitch and location feature changes of the syllables, and whether these effects differed between the two participant groups, who either could preselect one task-relevant feature or had to attend both features of syllables. A linear mixed-effects model (Table 1) revealed significant main effects of *retro-cue* (*β* = –0.076, *s.e.* = 0.006*, t*(39.13) = *–*12.56, *p* < 0.0001), and *feature type* (*β* = –0.034, *s.e.* = 0.016, *t*(38.20) = *–*2.93, *p* = 0.0057), but no significant interaction effect (*p* = 0.87). As expected, participants were significantly faster at judging featural changes in probe syllables when they received valid than neutral retro-cues. Also, participants were significantly faster at judging pitch than location changes of probe syllables (Fig. 2A).

**Table 1:** Statistical results of the linear mixed-effects model of the log-transformed response times (RTs) of correct trials

Predictors	*β*	*s.e*	*t*	*df*	*p*	95% CI
**Retro-cue**	**–0.076**	**0.006**	**–12.56**	**39.13**	< 0.0001***	**[–0.088, –0.064]**
**Feature type**	**–0.034**	**0.016**	**–2.93**	**38.20**	0.0057**	**[–0.057, –0.011]**
**Feature predictability**	**–0.14**	**0.038**	**–3.52**	**38.00**	0.0011**	**[–0.22, –0.062]**
Retro-cue × Feature type	0.0008	0.005	0.16	5277	0.87	[*–*0.009, 0.010]
**Retro-cue × Feature predictability**	**–0.016**	**0.006**	**–2.60**	**39.13**	0.013*	**[–0.027, –0.004]**
Feature type × Feature predictability	0.0012	0.012	0.11	38.2	0.92	[–0.021, 0.024]
Retro-cue × Feature type × Feature predictability	0.0029	0.0049	0.59	5277	0.56	[–0.007, 0.012]

*p < 0.05*,* **p < 0.01*,* ***p < 0.001

The model also revealed significant effects of *feature-predictability* group (*β* = *–*0.14, *s.e.* = 0.038, *t*(38.00) = *–*3.52, *p* = 0.0011), and *retro-cue* × *feature-predictability* group interaction (*β* = *–*0.016, *s.e.* = 0.006, *t*(39.13) = –2.60, *p* = 0.013). These patterns indicate that listeners who could preselect a single to-be-probed feature during task performance were significantly faster compared with those who had to attend both features. Furthermore, faster responses with valid relative to neutral retro-cues was more pronounced in the feature-predictable group (*z* = 10.83,* p* < 0.0001) compared with those who had to attend both features (*z* = 6.97,* p* < 0.0001).

Figure 2B illustrates task performance accuracy of each condition. Both groups of participants exhibited above-chance performance in judging pitch and space changes during the task even in the baseline (i.e., neutral cue) condition (Table 2; all *t* values > 4.91, *p* values < 0.0001 from one-sample *t* tests against 50%). A generalized linear mixed-effects model (Table 3) revealed a marginal effect of *retro-cue* (*β* = 0.048 s*.e.* = 0.028, *z* = 1.73, *p* = 0.083) but significant effects of *feature type* (*β* = 0.63, *s.e.* = 0.039, *z* = 16.09, *p* < 0.0001) and their interaction (*retro-cue* × *feature type*: *β* = 0.068, *s.e.* = 0.028, *z* = 2.48, *p* = 0.013). These effects indicate that participants were generally more accurate in discriminating pitch than location changes. Also, post hoc tests indicated that valid retro-cues enhanced memory recall accuracy for pitch (*z* = 2.63,* p* = 0.0085), whereas no such benefit was observed for location changes (*z* = 0.60,* p* = 0.55).

**Table 2:** Mean (± *SD*) and 95% CI of performance accuracy (%) in each condition

	Pitch		Space	
Group	Valid	Neutral	Valid	Neutral
Feature-predictable	87.5 ± 0.06 [84.5, 90.5]	84.3 ± 0.07 [80.9, 87.7]	61.2 ± 0.07 [57.9, 64.5]	61.3 ± 0.08 [57.5, 65.0]
Feature-unpredictable	81.5 ± 0.10 [76.9, 86.0]	78.4 ± 0.09 [74.5, 82.4]	57.4 ± 0.07 [54.3, 60.6]	59.4 ± 0.05 [57.2, 61.5]

**Table 3:** Results of the generalized linear mixed-effects model of the trial-wise performance accuracy

Predictors	*β*	*s.e*	*z*	*p*	95% CI
Retro-cue	0.048	0.028	1.73	0.083^†^	[–0.006, 0.10]
**Feature type**	**0.63**	**0.039**	**16.09**	< 0.0001***	**[0.55, 0.71]**
**Feature predictability**	**0.14**	**0.049**	**2.83**	0.0047**	**[0.043, 0.24]**
**Retro-cue × Feature type**	**0.068**	**0.028**	**2.48**	0.013*	**[0.014, 0.12]**
Retro-cue × Feature predictability	0.020	0.028	0.70	0.48	[–0.035, 0.074]
**Feature type × Feature predictability**	**0.079**	**0.039**	**2.03**	0.042*	**[0.003, 0.16]**
Retro-cue × Feature type × Feature predictability	0.001	0.028	0.02	0.99	[–0.054, 0.054]

Type III ^†^*p* < 0.1, *p < 0.05, **p < 0.01, ***p < 0.001

The same model also revealed significant effects of *feature-predictability* group (*β* = 0.14, *s.e.* = 0.049, *z* = 2.83, *p* = 0.0047) and *feature type* × *feature-predictability* group interaction (*β* = 0.079, *s.e.* = 0.039, *z* = 2.03, *p* = 0.042), but no other significant interaction effects (*p* values > 0.48). As expected, the feature-predictable group showed higher performance accuracy than the feature-unpredictable group. However, this group difference was evident only for pitch judgments (*z* = 2.72, *p* = 0.0065) and not for syllable location judgments (*z* = 1.58, *p* = 0.11).

Notably, while participants performed above chance in all conditions, baseline performance (i.e., the neutral cue condition) was higher for pitch than for spatial judgments. This difference may reflect the fact that applying identical multipliers to each participant’s 50% threshold did not necessarily yield perceptually equivalent changes across the two feature dimensions. To determine whether this baseline discriminability difference influenced the relative benefits from valid (vs. neutral) retro-cues, we conducted a control analysis that included each individual’s baseline performance as a covariate along with its interactions with the retro-cue and feature-type factors. In this model (Table S1), we found that even after controlling for individual differences in discriminability, the retro-cue × feature type interaction remained significant (*β* = 0.15, *s.e.* = 0.047,* z* = 3.22, *p* = 0.0013). This confirms that the differential retro-cue benefits for pitch versus spatial features were not solely driven by baseline discriminability differences.

However, after controlling for baseline discriminability, the previously observed main effect of feature-predictability group and its interaction with feature type were no longer significant (Table S1). This suggests that the retro-cue and feature-type effects were comparable across the two groups, and that group-related performance differences were largely explained by individual variability in baseline performance. In addition, among participants who completed the post-experiment questionnaire (*n* = 33 out of 40), the two groups provided comparable ratings of their task understanding, as well as the perceived helpfulness and use of the valid retro-cues (Table S2). Thus, although the feature-unpredictable group showed overall lower accuracy than the feature-predictable group, these results suggest that this group difference was likely due to increased memory and attentional demands rather than the differences in task understanding or engagement.

**Fig. 3:**
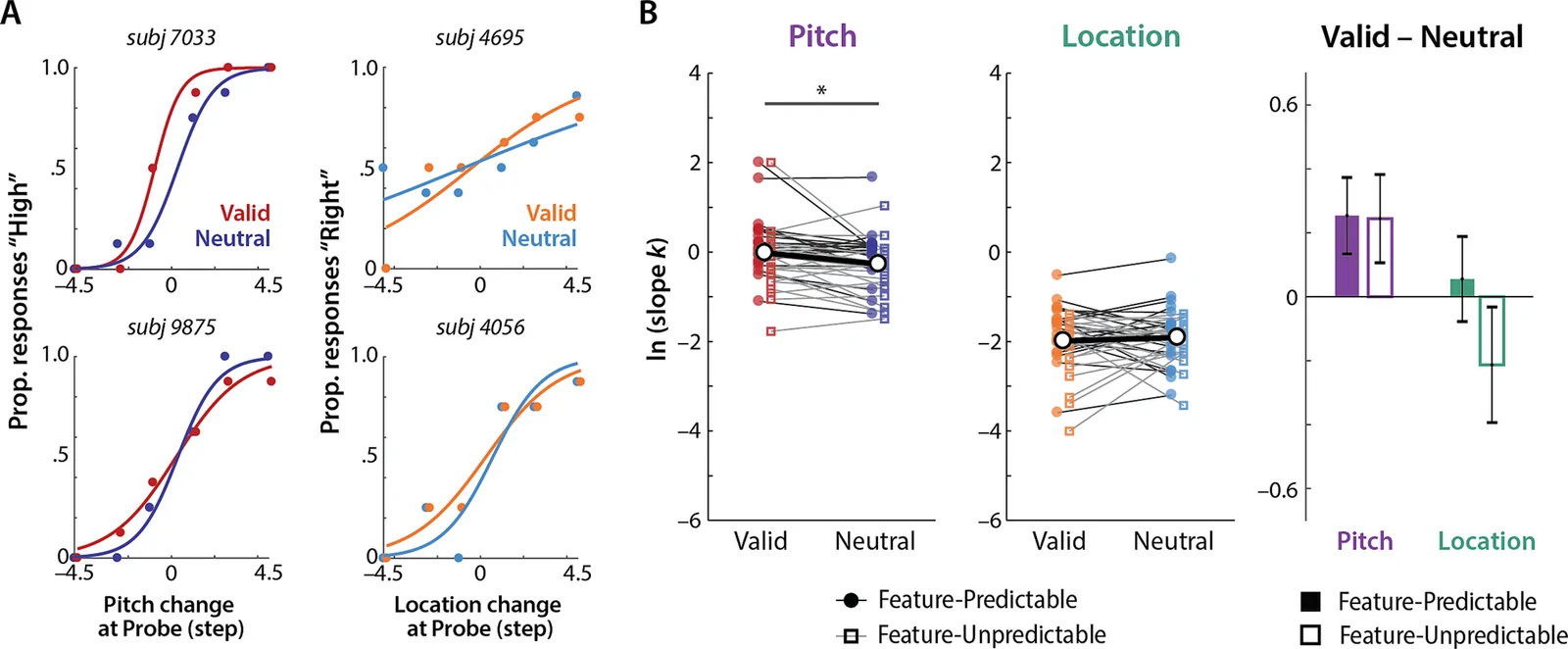
Perceptual precision estimates from psychophysical modeling results. **(A)** Exemplary psychophysical modeling fits of *n* = 4 representative individuals’ response patterns in judging changes in pitch (left) and location (right) dimensions in the valid and neutral retro-cue trials. Dots represent the individual’s proportion of responses as a function of respective featural change relative to the reference syllable sound heard during encoding. The lines represent model fits of the respective retro-cue × feature-type conditions. **(B)** Perceptual precision estimates (ln slope *k*) by feature type. Individual and group-average estimates of the valid and neutral retro-cue conditions (left) and the group average precision difference between conditions (valid minus neutral; right). Individual and mean grand average estimates are represented using the same illustration scheme in Fig. 2. *p < 0.05. (Color figure online)

We further examined whether valid retro-cues enhanced perceptual precision in recalling the pitch and location features of syllables held in memory. Using a psychophysical modeling approach (Bays & Husain, 2008; Zhang & Luck, 2008), we estimated fine-grained measure of perceptual precision for each participant, separately for each feature. A mixed-effects model on perceptual precision (log-transformed slope *k*) revealed no significant main effect of *retro-cues* (*β* = 0.043, *s.e.* = 0.038, *t*(38.0) = 1.12, *p* = 0.27), but significant effects of *feature type* (*β* = 0.90, *s.e.* = 0.066, *t*(38.0) = 13.74, *p* < 0.0001), and *retro-cue* × *feature type* interaction (*β* = 0.082, *s.e.* = 0.034, *t*(38.0) = 2.40, *p* = 0.022). Overall, participants recalled pitch with higher precision than location. Consistent with previous findings (Kumar et al., 2016; Lim et al., 2015, 2018, 2021), valid retro-cues enhanced precision for pitch relative to neutral cues (*z* = 2.43, *p* = 0.017); however, no such effect was observed for syllable location (*z* = 0.77, *p* = 0.44; Fig. 3B).

The model also revealed a significant effect of *feature-predictability* group (*β* = 0.18, *s.e.* = 0.062, *t*(38.0) = 2.84, *p* = 0.0073), but no significant interactions (*p* values > 0.35; Table 4). This pattern suggests that participants in the feature-predictable group demonstrated overall higher recall precision compared with those in the feature-unpredictable group. However, the pitch-specific precision enhancement from valid retro-cues (but absent for syllable location) was comparable regardless of participants’ awareness of the task-relevant feature.

**Table 4:** Statistical results of the perceptual precision estimate (ln slope *k*) from the psychophysical modeling fit

Predictors	*β*	*s.e*	*t*	*df*	*p*	95% CI
Retro-cue	0.043	0.038	1.12	38.0	0.27	[–0.032, 0.117]
**Feature type**	**0.90**	**0.066**	**13.74**	**38.0**	< 0.0001***	**[0.77, 1.03]**
**Feature predictability**	**0.18**	**0.062**	**2.84**	**38.0**	0.0073**	**[0.055, 0.299]**
**Retro-cue × Feature type**	**0.082**	**0.034**	**2.40**	**38.0**	0.022*	**[0.015, 0.15]**
Retro-cue × Feature predictability	0.035	0.038	0.91	38.0	0.37	[–0.04, 0.11]
Feature type × Feature predictability	0.037	0.066	0.57	38.0	0.57	[–0.091, 0.166]
Retro-cue × Feature type × Feature predictability	–0.032	0.034	–0.95	38.0	0.35	[–0.099, 0.035]

Type III *p < 0.05, **p < 0.01, ***p < 0.001

Using an approach analogous to the analysis of the performance accuracy measure, we constructed an additional control mixed-effects model to account for individual’s baseline perceptual precision (i.e., precision *k* parameter of the neutral cue trials) for both features. In this model (Table S3), the effect of *retro-cue* × *feature type* interaction remained highly significant (*β* = 0.23, *s.e.* = 0.044, *t*(91.33) = 5.20, *p* < 0.0001). Post hoc tests revealed a consistent pattern; valid retro-cues significantly enhanced pitch precision relative to neutral cues (*t*(78.7) = 4.60, *p* < 0.0001), whereas spatial location precision was reduced under valid compared with neutral cues (*t*(78.7) = –3.15, *p* = 0.0023). However, as in the accuracy analysis, this model no longer revealed any significant effects related to feature-predictability group (Table S3; all *p* values ≥ 0.13), suggesting that the overall group difference in recall precision was largely attributable to baseline differences in perceptual precision rather than to differences in the magnitude of retro-cue benefits. For the response bias estimate (*m*), we did not find any significant effects of the factors of interest (all *t* values < 1.48, *p* value*s* > 0.15).

## Discussion

Working memory and selective attention are closely intertwined (Awh et al., 2006; Chun, 2011; Gazzaley & Nobre, 2012). Within working memory, attention can be directed to internal representations, enhancing representational fidelity (Bays & Husain, 2008; Bays et al., 2011; Ma et al., 2014; Souza & Oberauer, 2016) and facilitating access to the features associated with the selected object (Backer et al., 2015; Czoschke et al., 2021; Dyson & Ishfaq, 2008; Peters et al., 2015). Here, we tested the predictions of an object-based attention framework (Alain & Arnott, 2000; Shinn-Cunningham, 2008; Shomstein & Yantis, 2002) within auditory working memory using a retro-cueing task. Specifically, we examined whether retrospective attention enhances mnemonic precision in an object-based manner by highlighting both pitch and spatial features of the attended auditory object, or whether such benefits differ across features.

We found that attention redirected to memory facilitated recall performance, replicating the beneficial effects of retrospective attention (Backer & Alain, 2014; Griffin & Nobre, 2003; Kumar et al., 2013; Lepsien & Nobre, 2007; Lim et al., 2015; Oberauer & Hein, 2012; Pertzov et al., 2013; Souza & Oberauer, 2016). In the present study, valid cues informed only the upcoming probe syllable category (i.e., object-level linguistic unit), while the task required participants to discriminate low-level features (i.e., pitch or spatial location) of the cued syllable. Nonetheless, listeners were significantly faster at judging featural changes with valid compared with neutral retro-cues. The beneficial effect on response speed was comparable for both features regardless of whether listeners were aware of the task-relevant feature (Fig. 2A). The consistent recall-speed benefits from attention-to-memory supports the view that stimulus-specific information is retained in working memory (Christophel et al., 2012; Harrison & Tong, 2009; Linke et al., 2011; Uluç et al., 2018), and that pitch and spatial location information of objects are preserved in auditory working memory (Czoschke et al., 2021; Fischer et al., 2024). Thus, although valid retro-cues provide information orthogonal to the feature dimension being probed, retrospective attention facilitates faster access to the cued memory item, prioritizing it for the upcoming task demands (Backer et al., 2020; Myers et al., 2017).

However, in contrast to the response speed, we found that recall accuracy and precision benefits from retrospective attention were selective for the pitch feature and absent for spatial location of the syllable object. This attentional enhancement in syllable pitch replicates previous findings showing that valid retro-cues lead to higher precision for recalling the pitch feature of attended auditory memory objects (Kumar et al., 2016; Lim et al., 2015, 2021). The current results extend this by demonstrating that pitch precision enhancement occurs even for the listeners who were unaware of the to-be-probed feature (Fig. 3B). This pattern indicates that although pitch does not necessarily constitute a primary feature to identify syllable objects (cf. Czoschke et al., 2021; Fischer et al., 2024; Kumar et al., 2013, 2016), redirecting attention to a specific auditory memory object reliably enhances the perceptual precision of its pitch feature even without directed attention to that feature.

In contrast, we found no comparable benefit from valid cues for the spatial feature. Interestingly, even among the feature-predictable group participants, who were aware that spatial location was task-relevant and could therefore prioritize this feature during encoding, valid retro-cues did not yield more precise recall of this feature (Fig. 3B). Moreover, the asymmetry between pitch and spatial location remained significant even after controlling for baseline performance differences between the two features (Tables S1, S3). Together, these findings suggest that the benefit of selective attention redirected to auditory memory is not necessarily uniform across multiple features of an auditory object. Instead, valid retro-cues enhanced the fidelity of pitch, but not spatial feature, regardless of whether listeners knew in advance which feature would be probed.

What factor might constrain the precision enhancement of spatial information? Our results suggest that despite the fact that both the space and pitch features are bound to the same auditory memory object, these features are stored and represented separately (Ahveninen et al., 2006; Alain et al., 2001; Arnott et al., 2004; Pasternak & Greenlee, 2005; Rauschecker, 1998). This is consistent with the idea that different features are closely tied to their dominant sensory modalities; particularly, processing spatial information is more compatible with the visual than the auditory modality (Fleming et al., 2024; Welch & Warren, 1980). As such, neuroimaging evidence also supports this view: processing spatial features—in both visual and auditory domains—recruits visual-biased network in the lateral frontal cortex subserving attention and working memory functions (Michalka et al., 2015; Noyce et al., 2017, 2022). Consequently, the spatial feature may be bound relatively weakly to auditory objects (Delogu et al., 2014; Jackson, 1953; Kubovy & Van Valkenburg, 2001; Maybery et al., 2009), making it difficult to access and hence less amenable to enhancement when an auditory item was attentionally reselected from memory. Furthermore, because the effective use of informative valid retro-cues requires additional cognitive resources (e.g., Griffin & Nobre, 2003; Lim et al., 2015; Lin et al., 2021; van Moorselaar et al., 2015), the inherent difficulty in accessing the spatial feature of the auditory object may further limit the effective, but cognitively costly use of informative cues.

In contrast, pitch may function more like an object-identity (“what”) feature, which is automatically encoded and more readily accessed from auditory memory objects (Delogu et al., 2014; Maybery et al., 2009). Crucially, this pattern holds true even though pitch did not explicitly define object identity in the present study; participants memorized syllable sounds, and pitch variations did not alter their linguistic identities. Thus, the privileged retro-cue benefit for pitch cannot simply be attributed to pitch serving as the main defining cue of the object but may instead reflect intrinsic biases in auditory working memory. Supporting this view, Lim et al.’s (2021) fMRI study showed that more robust neural representations of the cued syllable category in the auditory cortex (i.e., superior temporal gyrus) predicted more precise recall of the syllable’s pitch. Also, directing selective attention to an auditory memory object reactivates its sensory-based memory traces in the auditory regions of the temporal cortex (Buchsbaum et al., 2005; Lim et al., 2021; Scott & Perrachione, 2019), which is intrinsically connected to the auditory-biased attention and working memory network in the lateral-frontal cortex, but not to the visual-biased network (Michalka et al., 2015; Noyce et al., 2017; Tobyne et al., 2017). Thus, reorienting attention to an auditory memory object may not enhance spatial precision because the reactivated auditory trace does not strongly interact with the visual-biased network important for processing spatial information.

Nevertheless, one possible explanation for the absence of spatial precision benefit might be due to sequential presentation of the to-be-encoded syllable sounds. To avoid auditory masking, we adopted sequential rather than simultaneous presentation; however, this could have discouraged the use of spatial information, an important cue for segregating simultaneously presented sound objects (Ihlefeld & Shinn-Cunningham, 2008; Shinn-Cunningham, 2005). Thus, our task could have weakened spatial representations in auditory memory. The other possibility is that the syllables were highly distinct in their non-spatial features (i.e., categories). Prior studies show that listeners rely less on the spatial cues when other features (like pitch and timbre) can reliably guide selective attention to a distinct auditory object (Bonacci et al., 2020). Thus, defining the sound objects mainly via highly distinctive syllable categories may have further reduced the utility of spatial cues. Although recent studies using sequential presentation of complex nonspeech tones have observed reliable maintenance of both pitch and location features (Czoschke et al., 2021; Fischer et al., 2024), it is possible that both sequential presentation and highly distinctive syllables in the current study could have down-weighted robust use and maintenance of spatial information.

Another possibility is that listeners encoded auditory spatial location in a relatively coarse or categorical format. Unlike pitch, which may be represented along a more continuous perceptual dimension, auditory spatial information may have been encoded in a gist-based or verbal format (e.g., “left” vs. “right”). In the present study, the two to-be-remembered syllables were always presented in opposite hemispaces to ensure that listeners discriminated the spatial shifts without relying on the unprobed syllable as a reference. However, this design may have encouraged categorical encoding of spatial location rather than maintaining precise spatial representations. Such coarse spatial coding may lack feature-level flexibility, making it less amenable to precision enhancement by retrospective attention. The present study cannot determine the specific strategies listeners used to encode the syllable sounds. Nonetheless, prior evidence indicates that listeners can accurately recall the spatial locations of auditory objects (Czoschke et al., 2021; Fischer et al., 2024), suggesting that auditory spatial information is not strictly limited to verbal or categorical representations.

A further consideration is that the observed asymmetry may have been influenced by differences in baseline discriminability between pitch and spatial features. In the present study, we applied equivalent multipliers to each participant’s 50% discrimination threshold to parametrically manipulate pitch and spatial changes. However, these manipulations may not have yielded equivalent perceptual changes across the two features during the main task. One contributing factor may be the asymmetric interdependence between pitch and location features, such that changes in pitch bias spatial judgments more strongly than spatial changes biasing pitch judgments (Delogu et al., 2014; Maybery et al., 2009). Because pitch and location co-varied simultaneously on every trial, pitch changes may have interfered more strongly with spatial judgments, resulting in lower baseline performance for spatial location. While such asymmetrical interdependence between pitch and location is typically documented when pitch serves as the primary feature defining the identity of nonspeech sounds (Delogu et al., 2014; Maybery et al., 2009), a similar pattern may have emerged in the present study even though pitch changes did not alter the linguistic identity of the syllable objects. Thus, features integrated within a higher-level auditory object like speech may still differ in their relative discriminability despite the use of equivalent threshold-based multipliers.

Spatial acuity gradients may have also contributed to differences in task difficulty. Because spatial precision typically decreases as sounds move away from the midline (e.g., Mills, 1958; Perrott & Saberi, 1990), equivalent physical changes in location may not have corresponded to equivalent perceptual changes across starting positions. Consistent with this possibility, although the effects of interest were fully counterbalanced across starting positions, spatial discrimination in the present study varied depending on whether the probed syllable originated at ±30° or ±60° azimuth (Fig. S1). This eccentricity effect was selective to spatial judgments; pitch discrimination remained unaffected by starting position.

Because the ability to discriminate sounds based on pitch versus space can be biased by the relative discriminability of each feature dimension (Woods et al., 2001), baseline discriminability differences could have contributed to the larger valid-cue benefit for pitch than for spatial location. Crucially, however, the asymmetric pattern of retrospective-attention benefits remained robust even after controlling for baseline discriminability differences between the two features (Tables S1, S3). This indicates that the present findings cannot be explained solely by such baseline differences. Nevertheless, a valuable direction for future studies would be to independently manipulate the two feature dimensions and estimate individual psychometric functions across the full range of changes for each feature and for each starting position. Equating these perceptual distances would help adjudicate whether retro-cue benefits depend primarily on relative discriminability, or whether pitch and spatial location differ more fundamentally in the extent to which they can be selectively enhanced within auditory memory objects.

Additionally, it is of note that to direct listeners’ attention to memory, we only presented object-level information (i.e., syllable category). However, Backer et al. (2015) demonstrate differences in neural mechanisms underlying auditory retrospective attention when listeners are cued with object-based (semantic) versus feature-based (spatial) information, despite the fact that both types of cues redirect attention to the same memory item. Based on this finding, it remains an open question for future research to determine whether a spatial retro-cue could overcome the observed constraint in enhancing mnemonic precision.

## Conclusion

There are ongoing debates on how auditory objects and their defining features are represented in working memory and how selective attention enhances the memory representations. Our present work aimed to answer these questions by elucidating how redirecting attention to auditory memory enhances representational fidelity of multiple features associated with the object. Here, we demonstrate that selective attention to an auditory memory item selectively improves representational fidelity of its pitch but not the spatial feature. This asymmetric pattern of precision enhancement reflects that the benefits of retrospective auditory attention are not necessarily uniform across features of an object, and rather depend on how different features are represented and prioritized within auditory working memory. Overall, our findings provide evidence that flexible enhancement of auditory mnemonic fidelity via retrospective attention is constrained and shaped by the intrinsic representational properties of the features associated with the selected object.

## Supplementary Information

Below is the link to the electronic supplementary material.

Supplementary file1 (PDF 176 kb)

## Data Availability

The data and materials for the experiment are available in the Open Science Framework repository (https://osf.io/zavrs/overview?view_only=db2e8d1e93d244dba44be09dfb1320e0). This experiment was not preregistered.

## Code availability

The code for processing the experimental data is available in the Open Science Framework repository (https://osf.io/zavrs/overview?view_only=db2e8d1e93d244dba44be09dfb1320e0).
