# Supplementary material for: Attention to memory: Asymmetric enhancement of mnemonic features in auditory working memory
Source: Psychon Bull Rev. 2026 Sep 10;33(7):234. doi: 10.3758/s13423-026-02991-8 (PMC13562329; doi:10.3758/s13423-026-02991-8)
Supplement: Supplementary file 1 [file 13423_2026_2991_MOESM1_ESM.pdf]

Supplementary Materials

Table S1. Statistical results of the generalized linear mixed-effects model of accuracy, accounting for individual's baseline performance in the neutral condition.

**Predictors**	***β***	***s.e.***	***z***	***p***
Retro-cue	0.039	0.029	1.35	0.18
Feature type	0.17	0.048	3.48	**0.0005*** **
Feature predictability	0.041	0.030	1.35	0.18
Baseline performance	0.57	0.051	11.21	**< 0.0001*** **
Retro-cue × Feature type	0.15	0.047	3.22	**0.0013** **
Retro-cue × Feature predictability	0.035	0.030	1.19	0.23
Feature type × Feature predictability	0.009	0.029	0.32	0.75
Retro-cue × Baseline performance	–0.11	0.051	–2.16	**0.031***
Feature type × Baseline performance	0.15	0.050	2.91	**0.0037** **
Retro-cue × Feature type × Feature predictability	0.010	0.028	0.35	0.73

*Note: Type-III *p < 0.05, **p < 0.01, ***p < 0.001; Baseline (neutral condition) performance was scaled and centered.*

Table S2. Summary of post-experiment questionnaire ratings (means and standard deviations) across participant groups.

Question	Feature Predictable	Feature-Unpredictable	*U*	*Z*	*p*
How well did you understand the task? (1 = very well; 6 = not at all)	1.71 ± 1.33	2.37 ± 1.83	116	0.68	0.49
How difficult did you find the task? (1 = very easy; 6 = very hard)	3.29 ± 0.91	3.37 ± 0.83	125.5	0.29	0.77
How helpful did you find the cues? (1 = very helpful; 6 = not helpful at all)	2.43 ± 1.02	2.47 ± 1.26	134	0.038	0.97
How often did you use the cue? (1 = always; 6 = never)	2.79 ± 1.05	2.11 ± 1.49	180.5	1.80	0.072

Note: Post-experiment questionnaire responses were obtained from 14 participants in the Feature-Predictable group and 19 in the Feature-Unpredictable group. Statistical reports are derived from Mann-Whitney U-tests.

Table S3. Statistical results of linear mixed-effects model of ln precision slope (k) parameter, accounting for individual's baseline performance in the neutral condition.

**Predictors**	***β***	***s.e.***	***t***	***df***	***p***
Retro-cue	0.043	0.038	1.12	41.24	0.27
Feature type	0.23	0.053	4.39	69.32	**< 0.0001*** **
Feature predictability	0.055	0.040	1.40	42.47	0.17
Baseline performance	0.84	0.055	15.36	89.16	**< 0.0001*** **
Retro-cue × Feature type	0.23	0.044	5.20	91.33	**< 0.0001*** **
Retro-cue × Feature predictability	0.060	0.038	1.56	42.85	0.13
Feature type × Feature predictability	–0.032	0.032	–0.99	37.76	0.33
Retro-cue × Baseline performance	–0.18	0.047	–3.93	95.37	**0.00016*** **
Feature type × Baseline performance	0.089	0.053	1.68	91.26	$0.096^{\dagger}$
Retro-cue × Feature type × Feature predictability	–0.020	0.024	–0.85	74.26	0.40

Note: Type-III † $p < 0.1$, ** $p < 0.01$, *** $p < 0.001$; Baseline (neutral condition) performance was scaled and centered.

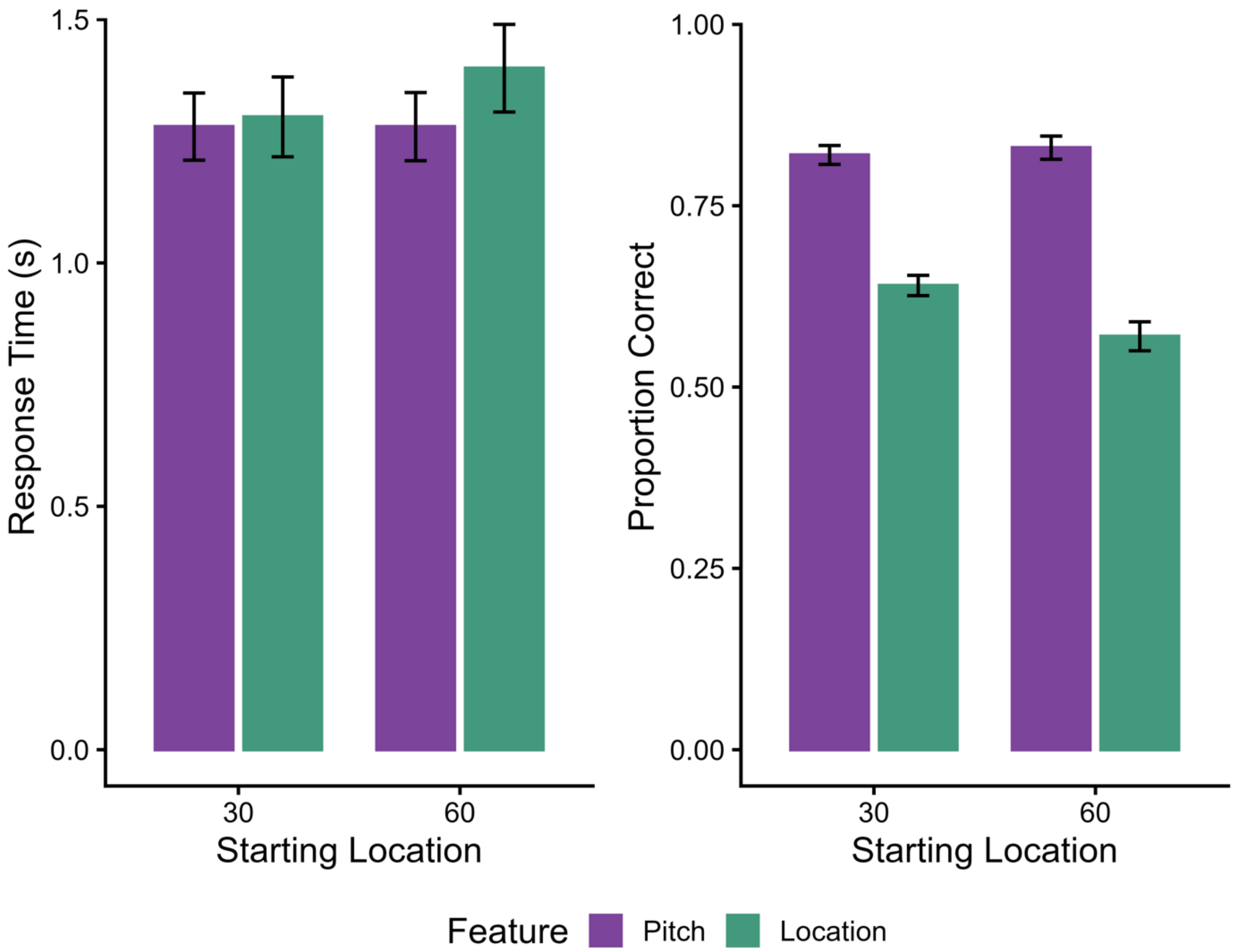

Figure S1. Average response times (correct trials only) and performance accuracy for each starting azimuth (±30 vs. ±60 degrees) by probed feature. Spatial performance was slower and less accurate at ±60-degree than at ±30-degree (RT: $t(39) = 3.11$, $p = 0.004$; accuracy: $t(39) = -5.70$, $p < 0.0001$), whereas pitch performance did not differ by starting position (*t*s <= 0.30, *p*s >= 0.76). The error bars represent ±1 standard error of the mean (SEM).
